# Multiuser Exercise-Based Telerehabilitation Intervention for Older Adults with Frailty: A Pilot Study

**DOI:** 10.3390/neurosci7010011

**Published:** 2026-01-13

**Authors:** Naoki Yamada, Itsuki Sato, Shoji Kinoshita, Atsushi Muraji, Seiki Tokunaga, Taro Naka, Ryo Okubo

**Affiliations:** 1 Department of Rehabilitation, University of the Ryukyus Hospital, Okinawa 901-2725, Japan; naka.taro1200@gmail.com; 2 Rehab for JAPAN Inc., Tokyo 102-0083, Japan; sato@rehabforjapan.com (I.S.); muraji@rehabforjapan.com (A.M.); tokunaga@rehabforjapan.com (S.T.); ryo.okubo@rehabforjapan.com (R.O.); 3 Department of Rehabilitation Medicine, The Jikei University School of Medicine, Tokyo 105-8461, Japan; kinoshita@jikei.ac.jp

**Keywords:** frailty, telerehabilitation, telehealth, quality of life, mHealth, aged, exercise

## Abstract

Objectives: This pilot study examined telerehabilitation, which has emerged as a crucial modality in light of recent global challenges such as the COVID-19 pandemic. We examined the effectiveness of a mobile health telerehabilitation intervention developed for older adults with frailty. Methods: Six participants received a telerehabilitation intervention (Rehab Studio) that included exercise training videos. The participants were aged ≥65 years, had no history of dementia or psychiatric disorders, and had mild-to-moderate care needs. For 1 month, the participants received 1 h live online rehabilitation sessions with real-time communication with rehabilitation specialists (physical therapists and occupational therapists: PTs/OTs). The quality of life (QoL) (EuroQol 5 dimensions 5-level [EQ-5D-5L] questionnaire) and self-rated health scores were recorded before and after the intervention, and the data were analyzed using paired t-tests to determine whether the service was effective. Results: Significant differences were found in the total EQ-5D-5L and self-rated health scores (*p* < 0.05). The mean EQ-5D-5L score increased from 0.63 ± 0.13 before the intervention to 0.77 ± 0.14 after the intervention (*p* = 0.010), while the mean self-rated health score increased from 66.0 ± 18.0 to 83.3 ± 10.3, respectively (*p* = 0.019). Conclusions: This study revealed that the mobile health telerehabilitation intervention is safe and can improve QoL for older adults with frailty. However, the effectiveness of the intervention needs to be further investigated in patients with poor performance in daily living activities. Telerehabilitation could help to reduce the burden of nursing care in aging societies with declining birthrates. However, given the extremely small sample size (*N* = 6), these *p*-values should be interpreted with considerable caution. Statistical significance in such a small sample does not provide strong evidence for population-level effects, and our findings should be regarded as hypothesis-generating rather than confirmatory.

## 1. Introduction

Following the COVID-19 pandemic, methods such as telerehabilitation have been studied in recent years as an alternative to traditional face-to-face rehabilitation. Telerehabilitation involves the use of communication devices such as smartphones, tablets, personal computers, and telerehabilitation resource guides to facilitate communication and exercise when caregivers and users are physically separated [1,2,3,4,5]. This type of therapy was designed according to the recommendations of physiotherapists. Patient-reported outcomes from randomized controlled trials have also confirmed positive experiences and satisfaction with telerehabilitation programs [6].

Although there are obvious cost advantages to providing telerehabilitation, whether this method is as effective as traditional methods for improving both clinical and quality-of-life (QoL)-related outcomes needs to be examined. Japan is considered a ‘super-aging society’; by 2025, 6.8 million baby boomers will be aged over 65 years, and the number of single-person households and baby boomers requiring care is expected to increase, according to the reports from the Ministry of Health, Labour and Welfare. Elderly people in Japan generally have a strong desire to live independently, and their future care needs will likely differ from those currently addressed by conventional nursing care services. According to a report published by the Ministry of Economy, Trade, and Industry in April 2018, by 2035, the number of people requiring nursing care will reach 10 million, and the gap between the supply and demand for nursing care personnel will grow to approximately 680,000 people in Japan. As a result, a substantial number of ‘nursing care refugees’ will be unable to access nursing care services. Given this context, it is crucial to construct social infrastructure that addresses the physical, cognitive, and emotional needs of the elderly and ensures that they are not socially isolated. Such infrastructure must also incorporate technologies that are currently not covered by Japan’s long-term care insurance (LTCI) system. The LTCI system includes seven levels of care: two levels of supportive care and five levels of nursing care. An individual can receive nursing care services if their certification results fall under one of the seven levels.

Exercise instructions for older adults using mobile technology have been suggested to improve physical activity, and older adults who have received telecare interventions have been reported to experience improvements in their QoL [7,8,9,10]. Although many general systematic and scope reviews of telerehabilitation have focused on elderly people, none have focused on the effectiveness of exercise-based mHealth or telerehabilitation for frail older adults [11,12,13]. Therefore, in this study, we aimed to investigate the QoL-related outcomes [14,15,16,17,18,19,20] of simultaneous telerehabilitation in multiple users.

### 1.1. Development of Interventions

**Figure 1:**
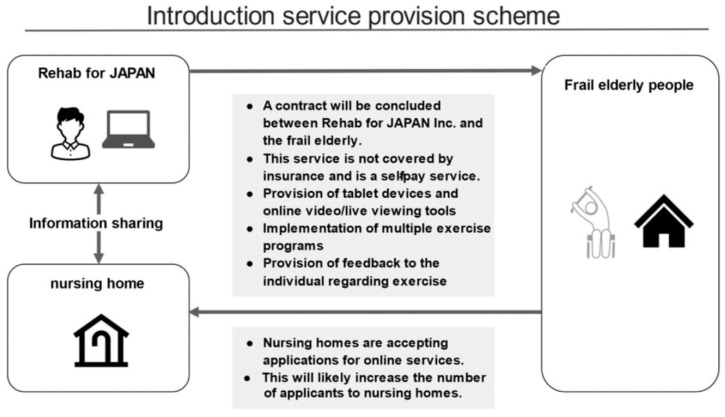
Overview of the telerehabilitation service provision.

In response to the increased need for telerehabilitation, a rehabilitation technology company has developed Rehab Studio, an exercise-based remote rehabilitation service that can be used by many frail elderly people simultaneously (Figure 1). The Rehab Studio platform uses a customized version of an online web conferencing system.

**Figure 2:**
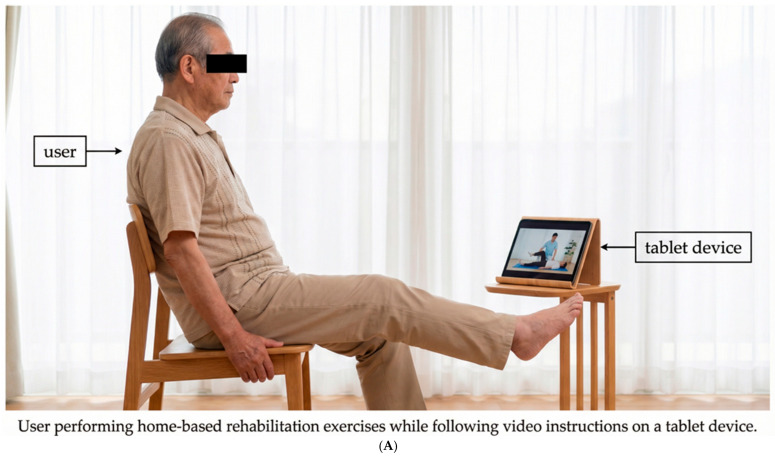

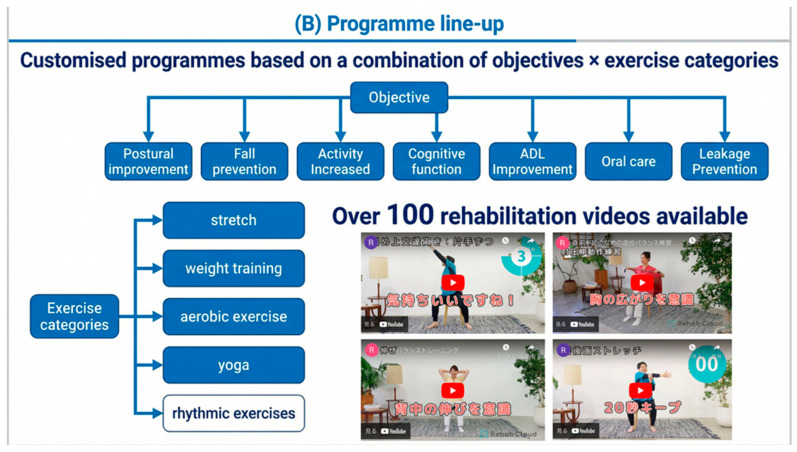
Home implementation of the telerehabilitation intervention.

This service allows the type and timing of exercises to be adjusted according to the user’s physical condition and preferences. The training videos can be viewed at any time and are selected by rehabilitation specialists according to the users’ current goals and physical condition so that the users can exercise daily (Figure 2).

The training videos include several exercises, such as upper and lower limb and trunk muscle strengthening exercises, and stretching, sitting, and standing balance exercises. A short video of approximately 5 min consists of three different exercises, while a video of 15–20 min consists of approximately 10 different exercises. More than 100 training videos and more than 2000 different exercises are available in this resource. Training videos can be selected according to different objectives such as ‘fall prevention’, ‘improving activity levels’, and ‘dementia prevention’.

Eligible persons receive weekly telerehabilitation and may also use a separate day service or other LTCI services. The training videos have no time constraints, and daily exercise opportunities can be created by using them as independent training on days when telerehabilitation and LTCI services are not scheduled. The effectiveness of this intervention was analyzed in this study.

Exercise Protocol Structure: Each 1 h live session follows a structured format: (1) warm-up phase (5–10 min): gentle stretching and breathing exercises; (2) main exercise phase (40–45 min): multicomponent exercises including resistance training for major muscle groups (upper/lower limbs, trunk), balance exercises (sitting and standing), functional mobility exercises, and flexibility/stretching exercises; and (3) cool-down phase (5–10 min): gentle movements and relaxation. Exercise intensity was individually tailored using a perceived exertion scale (modified Borg scale). Individualization and Feedback: PTs/OTs conducted initial assessments of each participant’s physical capabilities, limitations, and goals. Exercises were selected from the video library based on individual needs and LTCI care level. During live sessions, PTs/OTs provided real-time verbal cues for proper form and technique, encouragement and motivation, immediate correction of unsafe movements, and monitoring of fatigue levels with intensity adjustments as required. Between sessions, participants had access to pre-recorded exercise videos for independent practice and could rate session satisfaction on a five-point scale, which PTs/OTs reviewed to adjust subsequent sessions.

### 1.2. Pilot Test of Rehab Studio

Prior to the current study, short-term pilot tests of the service were conducted (first: May 2020, second: August 2020, third: November–December 2020, fourth: February 2021, and fifth: August–September 2022). Although the pilot sample sizes ranged from a few to over 100 people, testing allowed the intervention to be refined and improved. A care provider supplied the tablet terminals, online videos, and live viewing tools to frail elderly individuals, and physical therapists (PTs) and occupational therapists (OTs) directly conducted exercise programs for the participants. This system allowed the users to perform exercises while watching an exercise program. Physical and occupational therapists provided individual feedback to the users and communicated interactively online.

For the fifth pilot test, in August 2022, four frail elderly individuals (two men and two women) who used a daycare service were provided with online exercise instructions and communication opportunities for 1 month. These users were certified as requiring long-term care under Japan’s LTCI system (two users required long-term care level 1 and two users required support level 2). Two of the four users expressed a desire to continue using the service, even if they had to pay for it. They also shared the following comments about the service: ‘The instruction was very detailed and informative’, ‘I feel more positive mentally’, and ‘It is good to be able to interact with other people without going outside’.

## 2. Methods

### 2.1. Evaluation of the Intervention

**Figure 3:**
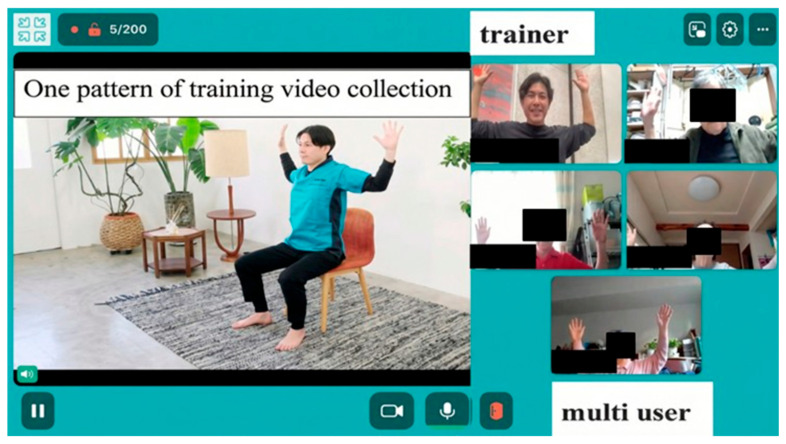
Presentation of the telerehabilitation intervention.

To preliminarily explore the potential effectiveness and feasibility of Rehab Studio in this pilot investigation, we selected 7 subjects. The inclusion criteria were as follows: those attending day care with support levels 1 or 2, and care level 1; absence of dementia; ability to walk alone with a cane or walker; and agreement to participate within the study timeframe. The exclusion criteria included a history of dementia or psychiatric disorders. Seven people agreed to participate, but one withdrew due to ill health, resulting in a total of six subjects who received 1 h live online rehabilitation sessions with multiple participants. The participants engaged with the sessions in a sitting position with real-time communication with rehabilitation specialists (PTs/OTs) using Rehab Studio (Figure 3).

**Figure 4:**
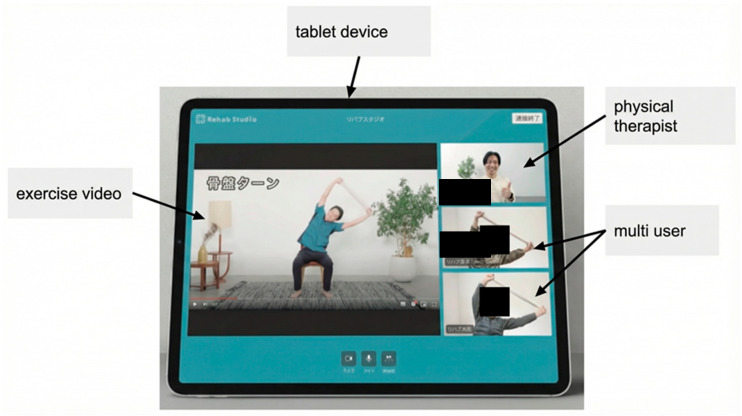
Dedicated tablet device configured for ease of use with the online web conferencing system ‘Whereby’ (Norway).

The Rehab Studio platform has the following advantages: After participating in the exercise program, users can enter their satisfaction levels on a five-point scale, and physical and occupational therapists can check the results; the system is based on participant experiments and designed such that even elderly people who are not comfortable with technology can easily participate in the program; and the system has been tested for several years. In addition, the service is continually updated based on feedback (on pricing, exercise program design, and safety considerations) from users who participated in the intervention over the last several years (Table 1). Rehab Studio uses a customized version of the online web conferencing system, ‘Whereby’ (Norway), which is specifically designed to allow older adults to participate without the requirement for email addresses or passwords (Figure 4).

To assess the effectiveness and safety of the Rehab Studio service, the EuroQol 5 dimensions 5-level (EQ-5D-5L) questionnaire was administered twice: before the intervention and 1 month after the intervention (Table 1). The EQ-5D-5L is a questionnaire with five items (walking, dressing, usual activities, pain/discomfort, and anxiety/blocked up) that are scored by the respondents on a five-point scale. We converted the responses obtained into a QoL score (maximum 1 point; minimum 0 points) using the conversion table of the Japanese version of the EQ-5D-5L.

### 2.2. Statistical Analysis

Given the small sample size (*n* = 6) and pilot nature of this study, we employed descriptive statistics and exploratory paired comparisons. Continuous variables (EQ-5D-5L and self-rated health scores) are presented as mean ± standard deviation (SD). Changes from baseline (pre-intervention) to post-intervention (1 month) were assessed using paired t-tests. The significance level was set at *p* < 0.05 (two-tailed). All statistical analyses were performed using JMP Pro version 18 (SAS Institute, Cary, NC, USA). We acknowledge that with a sample size of 6, statistical power is limited; therefore, these analyses should be considered to be exploratory. Furthermore, *p*-values derived from such a small sample may not provide reliable evidence for population-level effects, and statistical significance should not be interpreted as definitive proof of effectiveness.

## 3. Results

All six users exhibited improved EQ-5D-5L scores and self-rated health scores (see Table 1 for individual-level data). Given the small sample size (*n* = 6) and pilot nature of this study, these findings should be interpreted as preliminary and exploratory. The observed changes may not reliably reflect the variability or heterogeneity of the broader target population, and larger studies are required to confirm these preliminary observations. The mean EQ-5D-5L score increased from 0.63 ± 0.13 before the intervention to 0.77 ± 0.14 after the intervention (*p* = 0.010), while the mean self-rated health score increased from 66.0 ± 18.0 to 83.3 ± 10.3 (*p* = 0.019). All statistical tests were two-sided. Data were analyzed using paired t-tests. Significant differences were found in the total EQ-5D-5L and self-rated health scores (*p* < 0.05). The significance level was set at *p* < 0.05. Data were analyzed using JMP Pro version 18 (SAS Institute, Cary, NC, USA). The *p*-values presented should be interpreted with considerable caution, as the limited sample size substantially restricts our ability to draw reliable inferences about the broader population of frail older adults.

However, given the extremely small sample size (*n* = 6), these *p*-values should be interpreted with considerable caution. Statistical significance in such a small sample does not provide strong evidence for population-level effects, and our findings should be regarded as hypothesis-generating rather than confirmatory.

We present individual-level data in tabular format (Table 1) to provide complete transparency regarding the heterogeneity of responses. Given the extremely small sample size, graphical representations of individual trajectories were not included, as such visualizations might overemphasize patterns that may not be reproducible in larger samples.

## 4. Discussion

### 4.1. Main Findings and Clinical Significance

In this pilot study, we observed preliminary evidence suggesting that a one-month multiuser exercise-based telerehabilitation intervention led to statistically significant improvements that, while promising, require confirmation in larger studies in both EQ-5D-5L scores and self-rated health scores among all six frail older adult participants. The mean EQ-5D-5L score increased from 0.63 ± 0.13 to 0.77 ± 0.14 (*p* = 0.010), representing a 0.14-point improvement that exceeds the minimal clinically important difference (MCID) of 0.07–0.10 for the EQ-5D-5L in older populations [21,22]. The mean self-rated health score increased from 66.0 ± 18.0 to 83.3 ± 10.3 (*p* = 0.019), indicating a 26% improvement in participants’ subjective health perception. The EQ-5D-5L assesses five dimensions—mobility, self-care, usual activities, pain/discomfort, and anxiety/depression—capturing both physical and psychological components of health [23]. The improvements observed across these domains suggest that the intervention may have contributed to multidimensional well-being rather than isolated physical gains, which is particularly relevant for frail older adults whose health status is characterized by multisystem decline [24,25].

### 4.2. Social Engagement and Multiuser Format

The multiuser, group-based format represents a key distinguishing feature that may have contributed to the observed improvements. Previous studies have examined telerehabilitation in older adults with various conditions [7,14,15,16,17,26,27,28,29,30,31,32,33,34,35]. However, few have specifically focused on multiuser group formats for frail older adults in the Japanese long-term care context. Social isolation has been identified as a significant risk factor for mortality and functional deterioration in older adults, with effect sizes comparable to established risk factors such as smoking [36,37]. In the context of frailty, social isolation can create a vicious cycle where physical limitations reduce opportunities for social engagement, which in turn accelerates functional decline [38,39]. The real-time video conferencing format employed in our study allowed participants to see and interact with both therapists and other participants, creating a virtual community. Research has demonstrated that group-based exercise interventions for frail older adults not only improve physical function but also enhance social networks and reduce depressive symptoms [40,41]. Our multiuser telerehabilitation format may potentially disrupt the isolation–frailty cycle by providing both physical benefits through structured exercise and psychosocial benefits through shared online engagement.

COPD: chronic obstructive pulmonary disease; QOL: quality of life.

### 4.3. Technological Accessibility

A critical success factor for telerehabilitation is technological accessibility for older adults, many of whom have limited digital literacy [42,43]. The Rehab Studio platform was specifically designed to address these barriers by eliminating the requirement for email addresses or passwords and providing pre-configured tablet devices that automatically connect to scheduled sessions. These design considerations align with principles of age-friendly technology design [44] and address usability barriers that have been identified as major obstacles to telehealth adoption among older adults [45,46].

### 4.4. Exercise and Frailty Management

The exercise component aligns with evidence that structured physical activity can prevent and potentially reverse frailty in older adults [47,48]. Frailty is increasingly recognized as a dynamic state that can be modified through appropriate interventions [49]. Multicomponent exercise programs addressing strength, balance, and endurance have emerged as effective interventions for frailty prevention and management [50,51]. Our platform incorporated all three components through a library of exercises covering strengthening, stretching, and balance activities, with personalization critical for the heterogeneous frail older adult population [52].

### 4.5. Healthcare Delivery Context

Telerehabilitation gained unprecedented attention during the COVID-19 pandemic [53,54]; however, its value extends beyond pandemic-related necessity. Many older adults face barriers to accessing traditional rehabilitation services, including transportation difficulties, geographical remoteness, and physical limitations [55]. The multiuser format leverages the scalability advantages of telehealth while maintaining social engagement benefits. This model is particularly relevant in Japan’s healthcare system, which faces projected shortages of rehabilitation professionals due to population aging [56].

### 4.6. Study Limitations

This study has several important limitations. First, the small sample size (*n* = 6) substantially limited statistical power and generalizability [57,58]. While we observed statistically significant improvements, these findings should be considered preliminary and hypothesis-generating. The small sample size not only limits statistical power but also raises questions about the reliability and interpretability of *p*-values. In samples of this size, statistically significant results may arise from chance variation or may not be representative of the broader population, even when genuine effects exist. Therefore, our findings require confirmation in adequately powered studies before any definitive conclusions can be drawn. Second, the non-randomized design and absence of a control group limit causal inference [59,60]. Without a control group, we cannot definitively attribute improvements to the intervention rather than to placebo effects or temporal trends. Future studies should employ randomized controlled designs [61]. Third, the short intervention duration (one month) limited our ability to assess the sustainability of benefits [62]. Long-term follow-up assessments are required to evaluate the durability of effects [63]. Fourth, reliance on self-reported measures introduces potential response bias [64]. Future studies should include objective measures such as gait speed, grip strength, or accelerometer-measured physical activity [65,66]. Fifth, we did not systematically assess intervention adherence, which is critical for understanding effectiveness [67]. Sixth, our outcome assessment was limited to the EQ-5D-5L and self-rated health scores. While the EQ-5D-5L includes an anxiety/depression dimension, we did not employ validated mental health measures such as the Geriatric Depression Scale. This limits our ability to comprehensively assess the psychosocial benefits of the intervention, despite qualitative participant feedback suggesting improved mood and social connection. Finally, our study was conducted in a single urban area in Japan with participants recruited from established day care services. Therefore, the findings may not generalize to rural populations, other countries, or individuals not engaged with formal care services [68,69]. Moreover, cultural factors may influence both acceptability and effectiveness of telerehabilitation [70].

### 4.7. Future Research and Clinical Implications

Large-scale randomized controlled trials with adequate statistical power are required to definitively establish the effectiveness of multiuser exercise-based telerehabilitation for frail older adults [71,72]. These trials should include appropriate control groups and comprehensive outcome assessments, including quality of life, physical function, cognitive function, and healthcare utilization [73]. Research should also examine dose–response relationships to identify optimal intervention parameters [74], investigate subpopulation differences [75], and assess cost-effectiveness [76,77]. Despite limitations, this pilot study provides preliminary evidence supporting the feasibility, safety, and potential efficacy of multiuser exercise-based telerehabilitation for frail older adults. The positive outcomes add to evidence supporting policy reforms to expand insurance coverage for telerehabilitation services [78,79]. Moreover, the multiuser format offers a scalable model that could help address workforce shortages and improve access to rehabilitation services [80].

## 5. Conclusions

This pilot study provides initial exploratory evidence suggesting the potential feasibility and preliminary efficacy of a one-month multiuser exercise-based telerehabilitation intervention for improving quality of life in frail older adults. All six participants showed improvements in EQ-5D-5L and self-rated health scores, and the intervention was safe, with no reported adverse events. However, the small sample size, absence of a control group, short intervention duration, and reliance on self-reported outcomes limit conclusions regarding causality and generalizability. Provided our findings are confirmed in larger, methodologically rigorous trials, multiuser telerehabilitation may help to address challenges such as limited healthcare resources, workforce shortages, and increased demand for rehabilitation services in aging societies. By enabling healthcare providers and older adults to collaborate in virtual spaces, these technologies have the potential to improve health outcomes, enhance access to care, reduce isolation, and provide continuous support. With continued research, refinement, and policy support—pending confirmation through larger randomized controlled trials—multiuser exercise-based telerehabilitation might potentially become a useful complementary component of comprehensive care for frail older adults, complementing traditional in-person services and helping older adults to maintain independence, function, and quality of life as they age. New telemedicine, telerehabilitation, and mHealth technologies for elderly people can improve their QoL. Multiuser telerehabilitation could address problems such as limited healthcare resources and increased demand for rehabilitation services. By allowing healthcare providers, caregivers, and older adults to collaborate in real or virtual spaces, these new technologies can improve health outcomes and provide continuous support to users.

## Figures and Tables

**Table 1:** EQ-5D-5L scores before and 1 month after the start of the intervention for frail elderly patients *n* = 6).

No	1	2	3	4	5	6
Age	67	82	80	80	82	81
Sex	Male	Female	Female	Female	Female	Female
Disease name	OPLL, LDH	Hip osteoarthritis	LDH, DM	SAH	LCS, Lumbar kyphosis	Lumbar compression fracture
Nursing care certification	Needs level 2 support	Needs level 2 support	Needs level 2 support	Needs level 1 support	Needs level 2 support	Needs level 2 support
EQ-5D-5L score pre-intervention	0.55	0.76	0.52	0.48	0.82	0.66
EQ-5D-5L score post-intervention	0.60	0.78	0.73	0.61	1.00	0.88

EQ-5D-5L: EuroQol 5 dimensions 5-level quality of life scale; OPLL: ossification of the posterior longitudinal ligament; LDH: lumbar disc herniation; DM: diabetes mellitus; SAH: subarachnoid hemorrhage; and LCS: lumbar spinal canal stenosis.

## Data Availability

The datasets generated during and/or analyzed during the current study are available from the Japan medical association Database of clinical MEdicine (J-DOME).
